# History of dating violence and the association with late adolescent health

**DOI:** 10.1186/1471-2458-13-821

**Published:** 2013-09-10

**Authors:** Amy E Bonomi, Melissa L Anderson, Julianna Nemeth, Frederick P Rivara, Cynthia Buettner

**Affiliations:** 1 Human Development and Family Science, The Ohio State University, Columbus, OH, USA; 2 Human Development and Family Studies, Michigan State University, East Lansing, MI, USA; 3 Group Health Research Institute, Seattle, WA, USA; 4 College of Public Health, The Ohio State University, Columbus, OH, USA; 5 School of Public Health and Community Medicine and Department of Pediatrics, University of Washington, Seattle, WA, USA; 6 Seattle Children’s Hospital, Seattle, WA, USA

**Keywords:** Adolescents, Adolescent sexual behavior, Dating violence, Depression, Eating disorders

## Abstract

**Background:**

The present investigation expands upon prior studies by examining the relationship between health in late adolescence and the experience of physical/sexual and non-physical dating violence victimization, including dating violence types that are relevant to today’s adolescents (e.g., harassment via email and text messaging). We examined the relationship between physical/sexual and non-physical dating violence victimization from age 13 to 19 and health in late adolescence/early adulthood.

**Methods:**

The sample comprised 585 subjects (ages 18 to 21; mean age, 19.8, SD = 1.0) recruited from The Ohio State University who completed an online survey to assess: 1) current health (depression, disordered eating, binge drinking, smoking, and frequent sexual behavior); and 2) dating violence victimization from age 13 to 19 (retrospectively assessed using eight questions covering physical, sexual, and non-physical abuse, including technology-related abuse involving stalking/harassment via text messaging and email). Multivariable models compared health indicators in never-exposed subjects to those exposed to physical/sexual or non-physical dating violence only. The multivariable models were adjusted for age and other non-dating abuse victimization (bullying; punched, kicked, choked by a parent/guardian; touched in a sexual place, forced to touch someone sexually).

**Results:**

In adjusted analyses, compared to non-exposed females, females with *physical/sexual dating violence victimization* were at increased risk of smoking (prevalence ratio = 3.95); depressive symptoms (down/hopeless, PR = 2.00; lost interest, PR = 1.79); eating disorders (using diet aids, PR = 1.98; fasting, PR = 4.71; vomiting to lose weight, PR = 4.33); and frequent sexual behavior (5+ intercourse and oral sex partners, PR = 2.49, PR = 2.02; having anal sex, PR = 2.82). Compared to non-exposed females, females with *non-physical dating violence only* were at increased risk of smoking (PR = 3.61), depressive symptoms (down/hopeless, PR = 1.41; lost interest, PR = 1.36), eating disorders (fasting, PR = 3.37; vomiting, PR = 2.66), having 5+ intercourse partners (PR = 2.20), and having anal sex (PR = 2.18). For males, no health differences were observed for those experiencing *physical/sexual dating violence* compared to those who did not. Compared to non-exposed males, males with *non-physical dating violence only* were at increased risk of smoking (PR = 3.91) and disordered eating (fasting, using diet aids, vomiting, PR = 2.93).

**Conclusions:**

For females, more pronounced adverse health was observed for those exposed to physical/sexual versus non-physical dating violence. For both females and males, non-physical dating violence victimization contributed to poor health.

## Background

Approximately 36 percent of males and 44 to 88 percent of females experience dating violence victimization across the adolescent/young adult period [1,2]. Among females, studies have shown an association between having a history of *physical and/or sexual* dating violence victimization and poor health during adolescence—including depression [3-5]; anxiety and stress symptoms [6]; suicide ideation and/or attempts [5,7-11]; smoking, alcohol and drug use [3,5,8,10]; disordered eating (e.g., using laxatives and/or vomiting to lose weight) [8,10,11]; contracting a sexually transmitted disease [12,13]; having multiple sex partners [14]; pregnancy [8,14]; and diminished quality of life [7]. Male victims of *physical and/or sexual* dating violence during adolescence are at increased risk of disordered eating [4,5]; anxiety, stress symptoms and depression [4-6]; suicidal ideation and/or attempts [4,5]; smoking, alcohol and drug use [4,5,10]; and diminished emotional well-being [11]. Both *physical and emotional types* of dating violence increase anxiety and depression in adolescent males and females [15].

A recent longitudinal study by Exner-Cortens and colleagues (2013) examined health in late adolescence/young adulthood by dating violence types (psychological violence only and physical and psychological violence together) experienced from age 12 to 18 [16]. Subjects who experienced both physical and psychological violence were at risk for poor health outcomes; exposed females had increased risk of depression symptoms, suicidal ideation, smoking, and adult violence victimization, and exposed males had increased risk of adult violence victimization. Females who experienced psychological violence only were also at increased risk of heavy episodic drinking and adult violence victimization, and exposed males were at risk of antisocial behaviors, suicidal ideation, marijuana use, and adult violence victimization. The findings from Exner-Cortens’ study support those from other studies showing an increased risk of violence re-victimization in late adolescence/young adulthood if experienced earlier in adolescence [1,2,17].

Despite the strengths of the Exner-Cortens’ study (longitudinal design, large sample affording a separate assessment of how violence types impacted health outcomes) [16], the violence assessment was limited. Namely, violence victimization was assessed using five questions (called names/insulted; sworn at; threatened with violence; pushed/shoved; and had something thrown that could hurt). The assessment did not cover the range of violence types (physical, sexual, and non-physical abuse) recommended for assessment by the U.S. Centers for Disease Control and Prevention [18-20], including violence types relevant to today’s adolescents, such as harassment/stalking via text messaging, email, and social media [21,22].

Studies of adults have more extensively parsed health effects by specific types of violence experienced in intimate relationships, including a consideration of the different violence types (physical, sexual, and non-physical abuse) recommended for assessment by the U.S. Centers for Disease Control and Prevention [18-20]. These studies have shown that adults who experience physical/sexual types of violence within intimate (e.g., dating, marital) relationships tend to have more pronounced adverse health impacts (e.g., depression, chronic disease) than adults who experience non-physical types of abuse only (e.g., controlling behavior, insults) [23-26]. Sexual violence has the most devastating impacts on the health of adult women, including an association with severe depressive symptoms, post-traumatic stress disorder, anxiety, difficulty sleeping, fair/poor health, physical/somatic symptoms, cigarette smoking, and problem drinking [25,27]. However, the experience of non-physical victimization only (in the absence of physical/sexual abuse) is also associated with adverse mental health in adult women and men [23-26]. For example, in Bonomi’s study of 3,429 women ages 18 to 64, women who experienced recent non-physical intimate partner violence only had significantly lower vitality and social functioning, and were more likely to have minor or severe depressive symptoms compared to non-abused women [24].

The present investigation expands upon prior studies by examining the relationship between health in late adolescence and the experience of physical/sexual and non-physical only (e.g., threats, controlling behavior) dating violence from age 13 to 19. Our study significantly adds to the literature on the health correlates of specific types of adolescent dating violence. Specifically, our study includes an expanded assessment of how dating violence types relate to health in late adolescence, including dating violence types that are relevant to today’s adolescents [22]. Particularly relevant in today’s society are the ways in which technology, such as text messaging, email and social media [21,22,28-39], has affected teen relationships, including violence occurring in those relationships. For example, cell phones and other electronic mechanisms provide pathways for increased monitoring and harassment of dating/romantic partners and may exacerbate jealousy [21]. Yet, little is known about how excessive monitoring through mechanisms such as cell phones or email relate to late adolescent health. Similarly, “hooking up,” which is a primary pathway to relationship formation among today’s adolescents [40], also presents unique challenges [40,41] by presenting a ripe context for unwanted/coerced sex. Related, our study examines the association between sexual violence through verbal coercion and/or physical force and health in late adolescence [42-44].

## Methods

### Sample

Study procedures were approved by the institutional review board of The Ohio State University, including procedures to ensure the confidentiality and anonymity of subjects’ responses (e.g., data were immediately stripped of identifying information). The *analytic sample comprised 585 subjects* ages 18 to 21 enrolled at The Ohio State University, recruited in two data collection efforts. Subjects completed a one-time only online survey to assess current health and retrospective dating violence histories from age 13 to 19 (described below). The recruitment procedures were as follows:

•*Study 1*, conducted from March, 2011 to April, 2011, involved randomly sampling 730 students from the registrar’s office, from a total of 32,716 students age 18 to 21 enrolled at Ohio State University [22]. Using students’ university email account, we sent a recruitment email, which included the study description and link to the online survey. Two follow-up reminders were sent by email, three and seven days after the initial email. The cumulative response rate at each recruitment email was as follows: initial email (31.6%, 231/730); second email (41.0%, 300/730); final email (46.7%, 341/730) [22]. Subjects who completed the survey were credited $20 to their university account. Of the 341 subjects who completed the survey, 44 were excluded because they were older than age 21 (n = 7) or because they never had a dating partner from age 13 to 19 (n = 37). After these exclusions, the eligible analytic sample *comprised 297 subjects* who had a dating partner from age 13 to 19 (n = 190 females; n = 107 males).

•*Study 2*, conducted from May, 2011 to March, 2012, involved recruiting students ages 18 to 21 enrolled in four undergraduate Human Development and Family Science courses at The Ohio State University. Through an introductory email which included the study description and survey link, instructors offered completing the survey as extra credit coinciding with the coverage of relationship-related topics in class. A total of 311 students completed the survey; according to the class rosters, this represented 98% of eligible students. Of the 311 students, the eligible analytic sample *comprised 288 subjects* who had a dating partner from age 13 to 19 (n = 255 females and 33 males). The over-distribution of females in the sample is consistent with the gender distribution in the Human Development and Family Science undergraduate program.

•Our current analysis combined the two samples, *totaling 585 subjects* (n = 297 from the first study plus 288 from the second study). The over-distribution of females in Study 2 (recruited from undergraduate classes) compared to Study 1 (recruited through the university registrar) represented the main difference between the two samples, with otherwise similar characteristics between the two samples due to our narrow eligibility criteria (e.g., subjects had to be between the ages of 18 and 21).

### Survey

#### Health and health behaviors

To reduce response bias, subjects were first asked about health before they were asked about dating violence victimization. Asking subjects details about dating violence first, which could be a traumatic experience, could potentially cause bias in their responses to the health items; specifically, subjects might provide lower health ratings if the experience of completing the dating violence questions was traumatic [24].

•Depressive symptoms over the last two weeks were assessed using two questions from the nine-item Patient Health Questionnaire [45], which have a sensitivity of 74% for detecting depressive symptoms among adolescents relative to the Diagnostic and Statistical Manual of Mental Disorders [46]. The two questions included:

○ Having little interest in doing things;

○ Feeling down/hopeless.

•The two depression questions were scored as separate items, not as a composite measure; the response options for each question ranged from 0 (not at all) to 3 (nearly every day). In the analysis, the item responses were collapsed into a binary category (0 = not at all; 1 = experienced symptoms on any day).

•Unhealthy/disordered eating behaviors were assessed using three questions from the Youth Risk Behavior Surveillance System [47], including whether subjects ever:

○ Fasted, vomited, or took diet aids to lose weight (each its own separate question).

•Response options to the questions were binary (yes/no).

•Binge drinking and smoking were assessed using questions from the Youth Risk Behavior Surveillance System [47], including:

○ Ever smoked daily for 30 or more days;

○ Had 5 or more drinks on six or more days over last month.

•Response options to the questions were binary (yes/no).

•Sexual behavior was assessed by asking about subjects’ ever engagement (yes/no) in vaginal/penile intercourse, oral sex, and anal sex, and the number of partners they engaged in the sexual activity with (subjects reported the number of partners). The cut point for frequent oral and vaginal sex was defined as having five or more partners. Anal sex was less common, so the definition included having had any anal sex. While these measures are likely to indicate sexual health risk from sexual transmitted infections and unwanted pregnancy, they may not constitute equivalent risk.

#### Relationship and dating violence histories

We used a method similar to the timeline follow-back interview to assess dating violence histories retrospectively from age 13 to 19 [48-50]. We previously used this method to document domestic violence and child abuse histories in more than 4,000 women and men [24-26,51-57]. While retrospective dating violence assessment may result in mis-estimation of abuse due to recall bias [58,59], retrospective assessment is the field’s standard for capturing adolescent dating violence experiences and our assessment method used memory prompts to facilitate recall. The timeline follow-back interview method has been used extensively to capture other risky health behaviors, such as drug and alcohol use [48-50]. First, to establish relationship histories, subjects were asked whether they had a dating, romantic or sexual partner between age 13 and 19; this could include a boyfriend/girlfriend, someone the subject liked romantically or was involved with sexually but did not consider to be a boyfriend or girlfriend, or someone the subjected “hooked up with” [22]. They were then asked specific details about their three most recent partners, starting with their most recent partner, including the partner’s gender, the age the relationship began and ended, and the partnership type (e.g., boyfriend/girlfriend) [22,60]. We used memory prompts, such as asking the subject to remember the year they were in high school, to facilitate recall of the age that a relationship began and ended. For operational practicality, we asked details about subjects’ three most recent partners [22]. After we asked subjects detailed questions about their three most recent partners, we asked about the total number of partners subjects had beyond those three from age 13 to 19.

After information about subjects’ relationship history was gathered, dating violence victimization was assessed retrospectively using eight questions covering the three core conceptual areas of intimate (including dating) violence (physical, sexual, and non-physical) outlined by the Centers for Disease Control and Prevention [18-20] (Table 1):

**Table 1:** Dating violence questions (Has any partner you’ve been involved with between ages 13 and 19 ever …)

**Physical/sexual**	
	…hit, slapped, or physically hurt you on purpose?
	… pressured you to participate in sexual activities by begging or arguing with you, or by threatening to end your relationship?
	… pressured you to participate in sexual activities by threatening you with physical force (i.e., twisting your arm or holding you down)?
**Non-physical**	
	…threatened to hit or slap you, to spread rumors about you, to destroy something belonging to you, or to harm you in some other way?
	…tried to control your behavior by always checking up on you, telling you who your friends could be, or telling you what you could do and when?
	…called you names, put down your looks, or said things to hurt your feelings on purpose?
	…shouted, yelled, insulted, or sworn at you?
	…made unwanted phone calls, send unwanted text messages, emails, or gifts, or showed up in person and waited for you when you didn’t want them to?

Our eight questions were adapted from the CDC’s Youth Risk Behavior Surveillance System [47], Foshee and Swahn’s studies [61-63], and Coker’s dating violence survey currently being administered in a CDC-funded intervention study (unpublished data, personal communication with Dr. Coker). Additionally, our questions included newer forms of dating violence/abuse, including harassment/stalking through text messaging and email. Initial validity data from the eight dating violence questions we used in the present study were presented at the Women’s Health Congress in Washington, D.C. in March 2013 [64], and the validation manuscript is undergoing peer review; in brief, a confirmatory factor analysis of the eight dating violence questions showed that the questions loaded onto the hypothesized conceptual abuse factors (physical, sexual and non-physical abuse) [65].

For each question in Table 1, subjects were asked whether they ever experienced dating violence between age 13 and 19. Subjects who responded with “yes” were considered exposed to that abuse type. We created the following exposure groups based on prior studies that have conceptually and empirically examined physical and sexual violence within a single category [23,24,27,66], and psychological abuse only in a separate category [16,23,24,67].

•Physical and/or sexual dating violence exposure included subjects who reported experiencing physical and/or sexual types of dating violence. Subjects in this group could also have exposure to non-physical abuse. We included exposure to sexual pressure involving either (or both) *verbal* and *physical* coercion, as verbally coerced sexual acts have been shown to have lasting trauma for victims [25,43].

•Non-physical only dating violence exposure included subjects who were not exposed to physical/sexual dating violence, but who reported experiencing any of the non-physical dating violence types listed in Table 1. For ease in reporting, this group is referred to as the “non-physical TDV” group.

•Non-exposed subjects included those who reported never experiencing physical, sexual, or non-physical types of dating violence from age 13 to 19.

#### Other (non-dating) abuse exposures

Using three questions from the Centers for Disease Control [47,68], we asked about 1) whether subjects had ever been bullied between ages 13 and 19 (1 question); and 2) whether subjects experienced other types of abuse before age 18, including being punched, kicked, choked, or receiving a more serious physical punishment from a parent or other adult guardian (1 question) and being touched in a sexual place or being forced to touch another person when they did not want to (1 question).

### Analysis

All analyses were gender stratified. Chi-square tests were used to compare health indicators for subjects who reported any dating violence victimization with those who reported no victimization. Generalized linear models with a log link and robust sandwich variance estimators were used to obtain prevalence ratios (PRs) for each dichotomous health indicator for exposed compared to unexposed subjects, using a modified Possion regression approach [69]. Logistic regression models were not used because the health outcomes were not rare, and the odds ratios from these models would not closely approximate relative risks (or equivalently, prevalence ratios). The analyses investigated the effects of the type of dating violence experienced (physical/sexual versus non-physical only) on health indicators; specifically, regression coefficients compared health indicators in subjects with physical/sexual dating violence exposure compared to never-exposed subjects, and compared those with non-physical only dating violence compared to never-exposed subjects. We fit unadjusted and adjusted models; the adjusted models were adjusted for age, bullying victimization from age 13 to 19, and other non-dating physical and sexual abuse before age 18 (see Methods section and footnote in Table 2 for definitions)—all of which are theoretically linked and empirically associated with health impairments [58,70-75]. Analyses were completed using Stata statistical software, version 12.0 [76].

**Table 2:** Characteristics of the study sample

	**Female**	**Male**
Total	N = 445	N = 140
Age, mean (SD)	19.8 (1.0)	19.8 (1.0)
Year in college		
Freshman	114 (25.8)	45 (32.1)
Sophomore	149 (33.7)	41 (29.3)
Junior	139 (31.5)	41 (29.3)
Senior	40 (9.1)	13 (9.3)
Missing	3	0
Race		
White	377 (85.1)	116 (82.9)
Black	26 (5.9)	12 (8.6)
Asian	24 (5.4)	11 (7.9)
Other	16 (3.6)	1 (0.7)
Missing	2	0
Sexual orientation		
Heterosexual	410 (92.3)	127 (90.7)
Bisexual	16 (3.6)	1 (0.7)
Homosexual	6 (1.4)	7 (5.0)
Asexual	12 (2.7)	5 (3.6)
Missing	1	0
Ever been bullied (age 13 to 19)		
No	247 (55.9)	78 (56.1)
Yes	195 (44.1)	61 (43.9)
Missing	3	1
Physical child abuse		
No	410 (92.8)	127 (91.4)
Yes	32 (7.2)	12 (8.6)
Missing	3	1
Sexual child abuse		
No	387 (88.0)	135 (97.1)
Yes	53 (12.0)	4 (2.9)
Missing	5	1

## Results

### Characteristics of the study sample

The average age of subjects was 19.8 years, 76% comprised females, and most were enrolled in their freshmen or sophomore year (Table 2). Consistent with the Ohio State University student population in general (http://undergrad.osu.edu/admissions/quick-facts.html), most subjects reported that they were White (85% for females, 83% for males) and over 90% were heterosexual. Approximately 44% of subjects had been bullied between age 13 and 19. The proportion of females and males who suffered non-dating physical abuse before age 18 (being punched, kicked, choked, or receiving a more serious physical punishment from a parent or other adult guardian) was 7.2% and 8.6%, respectively. The proportion of females and males who were touched in a sexual place or forced to touch another person when they did not want to before age 18 was 12% and 2.9%, respectively.

### Prevalence of dating violence victimization

Table 3 shows the prevalence of dating violence victimization among females and males. A total of 67.4% of females and 57.1% of males reported dating violence victimization from age 13 to 19.

**Table 3:** Prevalence of dating violence victimization

	**Female (N = 445)**			**Male (N = 140)**		
	**# Reporting abuse**	**Prevalence (95% CI)**	**RSE†**	**# Reporting abuse**	**Prevalence (95% CI)**	**RSE†**
**Physical/sexual**						
Physical	25	5.6 (3.5, 7.8)	19.4	17	12.1 (6.7, 17.6)	22.8
Sexual – verbal coercion	112	25.3 (21.2, 29.3)	8.3	13	9.4* (4.5, 14.2)	26.5
Sexual – physical force	24	5.4 (3.3, 7.5)	19.9	1	0.7* (0.0, 2.1)	100
**Any physical/sexual**	126	28.4 (24.2, 32.6)	7.8	27	19.3 (12.7, 25.9)	17.7
**Non-physical**						
Threaten	53	12.0 (9.0, 15.0)	13.0	14	10.1* (5.0, 15.1)	25.4
Controlling behavior	124	27.9 (23.7, 32.1)	7.7	29	20.7 (14.0, 27.5)	16.5
Put down, name calling	152	34.3 (29.9, 38.7)	6.7	25	17.9 (11.5, 24.2)	18.6
Yell, swore, insulted	211	47.6 (43.0, 52.3)	5.0	57	40.7 (32.5, 48.9)	10.1
Unwanted calls, texts, visits	128	28.9 (24.7, 33.1)	7.6	36	25.7 (18.4, 64.7)	14.3
**Any non-physical**	287	64.6 (60.2, 69.1)	3.6	79	56.4 (48.2, 64.7)	7.4
**Any TDV**	300	67.4 (63.0, 71.8)	3.3	80	57.1 (48.9, 65.4)	7.3

† RSE = Relative Standard Error (standard error divided by the mean, expressed as a percentage).

*This estimate should be used with caution. Estimates with RSE > 25% are considered unreliable.

A total of 28.4% of females and 19.3% of males experienced physical and/or sexual violence; sexual violence experienced by females and males was mostly verbally coerced (25.3%, 9.4%) rather than physically forced (5.4%, 0.7%). Non-physical dating violence victimization occurred more frequently than physical/sexual violence, with 64.6% of females and 56.4% of males indicating they experienced this type of dating violence. Specific estimates of the various types of non-physical abuse are provided in Table 3; for example, being yelled at, sworn at, or insulted was the most common type of non-physical abuse for females (47.6%) and males (40.7%).

### Bivariate analysis

In the bivariate analysis, for females, any dating violence victimization was associated with depressive symptoms (feeling down/hopeless, having little interest in doing things), disordered eating (taking diet aids, fasting, vomiting), smoking daily for 30+ days, and frequent sexual behavior (vaginal intercourse with 5+ partners, oral sex with 5+ partners, and anal sex with 1+ partner) (Table 4). For males, any dating violence victimization was associated with depressive symptoms (feeling down/hopeless), taking diet aids, and smoking daily for 30+ days.

**Table 4:** Bivariate associations between health indicators and any dating violence victimization

	**Female**			**Male**		
	**No TDV**	**Any TDV**	**p-value**	**No TDV**	**Any TDV**	**p-value**
	**N = 145**	**N = 300**		**N = 60**	**N = 80**	
Ever smoked daily for 30+ days						
No	141 (97.2)	265 (88.3)	0.002	57 (95.0)	67 (83.8)	0.038
Yes	4 (2.8)	35 (11.7)		3 (5.0)	13 (16.3)	
5+ drinks on 6 or more days in past 30 days						
No	127 (87.6)	249 (83.0)	0.21	49 (81.7)	60 (75.0)	0.35
Yes	18 (12.4)	51 (17.0)		11 (18.3)	20 (25.0)	
**Depression (past 2 weeks)**						
Have little interest in doing things						
No	95 (65.5)	137 (45.7)	<0.001	39 (65.0)	44 (55.0)	0.23
Yes	50 (34.5)	163 (54.3)		21 (35.0)	36 (45.0)	
Feel down, hopeless						
No	107 (73.8)	166 (55.3)	<0.001	46 (76.7)	47 (58.8)	0.026
Yes	38 (26.2)	134 (44.7)		14 (23.3)	33 (41.2)	
**Disordered eating (ever)**						
Fasted 24+ hours to lose weight						
No	138 (95.2)	238 (79.3)	<0.001	57 (95.0)	71 (88.8)	0.19
Yes	7 (4.8)	62 (20.7)		3 (5.0)	9 (11.3)	
Taken diet aids to lose weight						
No	126 (86.9)	231 (77.3)	0.016	57 (95.0)	67 (83.8)	0.038
Yes	19 (13.1)	68 (22.7)		3 (5.0)	13 (16.2)	
Vomited to lose weight						
No	139 (95.9)	258 (86.3)	0.002	60 (100.0)	76 (95.0)	0.08
Yes	6 (4.1)	41 (13.7)		0 (0.0)	4 (5.0)	
**Number of sexual partners**						
Intercourse						
<5	128 (89.5)	219 (75.0)	<0.001	52 (91.2)	66 (83.5)	0.19
5 or more	15 (10.5)	73 (25.0)		5 (8.8)	13 (16.5)	
Oral sex						
<5	124 (86.7)	224 (76.5)	0.012	50 (86.2)	65 (81.3)	0.44
5 or more	19 (13.3)	69 (23.6)		8 (13.8)	15 (18.8)	
Anal sex						
None	133 (92.4)	241 (80.9)	0.002	53 (89.8)	66 (83.5)	0.29
1 or more	11 (7.6)	57 (19.1)		6 (10.2)	13 (16.5)	

TDV = Teen dating violence.

### Multivariable analysis

There were few differences between the unadjusted models and the models adjusted for age and other abuse victimization (bullying; punched, kicked, choked by a parent/guardian; touched in a sexual place, forced to touch someone sexually). We report the results from the adjusted models (Table 5).

**Table 5:** **Multivariable associations between health indicators and dating violence types (physical/sexual and non-physical only)**^**±**^

**Among female respondents**					
	No TDV	Physical and/or sexual	Non-physical only	Physical/sexual vs. No TDV	Non-physical only vs. No TDV
	N = 145	N = 126	N = 174		
	n (%)	n (%)	n (%)	PR (95% CI)	PR (95% CI)
Ever smoked daily for 30+ days	4 (2.8)	17 (13.5)	18 (10.3)	3.95 (1.38, 11.3)	3.61 (1.25, 10.5)
5+ drinks on 6 or more days in past 30	18 (12.4)	18 (14.3)	33 (19.0)	1.05 (0.57, 1.96)	1.51 (0.89, 2.56)
**Depression (past 2 weeks)**					
Have little interest in doing things	50 (34.5)	81 (64.3)	82 (47.1)	1.79 (1.37, 2.34)	1.36 (1.03, 1.79)
Feel down, hopeless	38 (26.2)	69 (54.8)	65 (37.4)	2.00 (1.45, 2.76)	1.41 (1.01, 1.97)
**Disordered eating (ever)**					
Fasted 24+ hours to lose weight	7 (4.8)	33 (26.2)	29 (16.7)	4.71 (2.12, 10.5)	3.37 (1.53, 7.45)
Taken diet aids to lose weight	19 (13.1)	32 (25.6)	36 (20.7)	1.98 (1.17, 3.36)	1.59 (0.96, 2.65)
Vomited to lose weight	6 (4.1)	22 (17.6)	19 (10.9)	4.33 (1.72, 10.9)	2.66 (1.09, 6.51)
**Number of sexual partners**					
Intercourse (5 or more)	15 (10.5)	34 (27.4)	39 (23.2)	2.49 (1.43, 4.32)	2.20 (1.27, 3.80)
Oral sex (5 or more)	19 (13.3)	35 (28.2)	34 (20.1)	2.02 (1.22, 3.33)	1.53 (0.92, 2.54)
Anal sex (1 or more)	11 (7.6)	28 (22.6)	29 (16.7)	2.82 (1.46, 5.45)	2.18 (1.15, 4.13)
**Among male respondents**					
	No TDV	Physical and/or sexual	Non-physical only	Physical/sexual vs. No TDV	Non-physical only vs. No TDV
	N = 60	N = 27	N = 53		
	n (%)	n (%)	n (%)	PR (95% CI)	PR (95% CI)
Ever smoked daily for 30+ days	3 (5.0)	3 (11.1)	10 (18.9)	2.28 (0.50, 10.3)	3.91 (1.18, 13.0)
5+ drinks on 6 or more days in past 30	11 (18.3)	9 (33.3)	11 (20.8)	1.94 (0.92, 4.07)	1.18 (0.71, 2.46)
**Depression (past 2 weeks)**					
Have little interest in doing things	21 (35.0)	9 (33.3)	27 (50.9)	0.94 (0.50, 1.78)	1.43 (0.93, 2.20)
Feel down, hopeless	14 (23.3)	11 (40.7)	22 (41.5)	1.68 (0.88, 3.23)	1.69 (0.98, 2.92)
Disordered eating (ever)*	6 (10.0)	3 (11.1)	16 (30.2)	1.08 (0.29, 4.02)	2.93 (1.24, 6.95)
**Number of sexual partners**					
Intercourse (5 or more)	5 (8.8)	4 (14.8)	9 (17.3)	1.78 (0.52, 6.09)	2.09 (0.75, 5.81)
Oral sex (5 or more)	8 (13.8)	6 (22.2)	9 (17.0)	1.52 (0.60, 3.82)	1.14 (0.47, 2.73)
Anal sex (1 or more)	6 (10.2)	3 (11.1)	10 (19.2)	1.10 (0.29, 4.15)	1.90 (0.73, 4.96)

TDV = Teen dating violence.

PR = Prevalence ratio.

CI = Confidence interval.

* Fasted 24+ hours, taken diet aids, or vomited to lose weight; these categories were combined for males due to the small sample size.

± Analysis adjusted for age, and prior abuse [bullying victimization, physical and sexual (non-dating) abuse victimization before age 18, including being punched, kicked, choked, or receiving a more serious physical punishment from a parent or other adult guardian before age 18 (1 question) and being touched in a sexual place or being forced to touch another person when they did not want to before age 18 (1 question)].

For females, more pronounced adverse health was observed for those exposed to physical/sexual versus non-physical dating violence victimization. In analyses adjusted for age and non-dating abuse victimization (see definitions in Table 2), compared to non-exposed females, females with *physical/sexual dating violence victimization* were at increased risk of smoking for 30+ days (PR = 3.95); depressive symptoms (feeling down/hopeless, PR = 2.00; lost interest in activities, PR = 1.79); eating disorders (taking diet aids, PR = 1.98; fasting, PR = 4.71; vomiting, PR = 4.33); and frequent sexual behavior (having 5+ vaginal intercourse partners, PR = 2.49; having 5+ oral sex partners, PR = 2.02; having anal sex, PR = 2.82). Compared to non-exposed females, females with *non-physical dating violence only* were at increased risk of smoking for 30+ days (PR = 3.61), depressive symptoms (feeling down/hopeless, PR = 1.41; lost interest in activities, PR = 1.36), eating disorders (fasting, PR = 3.37; vomiting, PR = 2.66), having 5+ intercourse partners (PR = 2.20), and having anal sex (PR = 2.18).

In contrast, for males, no health differences were observed for those experiencing *physical/sexual dating violence victimization* compared to those who did not experience physical/sexual dating violence. Compared to non-exposed males, males with *non-physical dating violence only* were at increased risk of smoking (PR = 3.91) and disordered eating (fasting, taking diet aids, vomiting, PR = 2.93).

## Discussion

Compared to non-exposed females, females who experienced physical/sexual dating violence victimization from age 13 to 19 were at increased risk of depressive symptoms, disordered eating (taking diet aids, fasting, vomiting), smoking, and frequent sexual behavior (having 5+ vaginal intercourse and oral sex partners, having anal sex) in late adolescence. Compared to non-exposed subjects, females and males who experienced non-physical dating violence only were at increased risk of smoking in late adolescence; in addition, exposed males were at increased risk of disordered eating and females were at increased risk of depressive symptoms, disordered eating, having 5+ vaginal intercourse partners, and having anal sex. Our study findings are consistent with Exner-Cortens’ recent longitudinal study noting more pronounced health impacts in late adolescence/young adulthood among females who had some experience of physical violence victimization [16]. Namely, the Exner-Cortens’ study showed that females with physical violence victimization had increased risk of depressive symptoms, suicidal ideation, smoking, and adult violence victimization [16]. However, males in the Exner-Cortens’ study seemed more adversely impacted by the experience of psychological abuse only, namely, they were at risk for antisocial behaviors, suicidal ideation, marijuana use, and adult violence victimization. Our study and that of Exner-Cortens were both limited in that they were not able to determine the qualitative nature of the dating violence acts assessed.

Our findings for females are also consistent with findings from other studies showing associations between physical/sexual dating violence victimization and adolescent depression [3]; disordered eating [8]; and having multiple sex partners [14]. Silverman and colleagues (2001) found strong associations between physical and/or sexual violence victimization and laxative use (odds ratio = 3.2) and vomiting (OR = 3.7) among adolescent females [8]; likewise, in our study, females who were physically and/or sexually abused by a dating partner had increased risk of using diet aids (prevalence ratio = 1.98), vomiting (PR = 4.31), and fasting 24+ hours (PR = 4.71). Silverman (2004) also found significant associations between the experience of physical/sexual dating violence victimization and having three or more sexual partners in the last three months [14]; this is consistent with our finding that females exposed to physical/sexual dating violence victimization were at increased risk of having multiple intercourse and oral sex partners.

Our findings for females are also consistent with Holt and Espelage’s (2005) finding that adolescents exposed to non-physical dating violence are at increased risk of depression [15]. Females in our study who experienced non-physical dating violence only were at increased risk of feeling down/hopeless (prevalence ratio = 1.41) and having little interest in doing things (PR = 1.36) compared to non-exposed females. In addition, females and males in our sample who were exposed to non-physical dating violence only (in the absence of physical/sexual violence) were at increased risk of smoking, males were at increased risk of disordered eating (combined category of fasting, using diet aids, vomiting), and females were at increased risk of fasting, vomiting, having 5+ vaginal intercourse partners, and having anal sex; these findings add to the literature on the health impact of non-physical dating violence, including Exner-Cortens’ recent longitudinal study [16].

Prior studies of males have shown that the experience of physical and/or sexual dating violence is associated with increased risk of disordered eating [4,5]; post-traumatic stress symptoms, anxiety, and depression [4-6]; suicidal ideation and attempts [4,5]; smoking, alcohol and drug use [4,5]; and diminished emotional well-being [11]. Our findings for males did not support findings from prior studies; we found no significant associations between the experience of physical/sexual dating violence victimization and the health indicators measured in our study. The sample size for males in our study was small, which reduced statistical power. As well, differences in the way constructs were operationalized across studies could potentially account for differences in findings.

Our study had limitations. First, generalizability is compromised due to our sample of predominantly White subjects enrolled at a large Midwestern university. While the racial/ethnic distribution of our sample mirrors that of the university (http://undergrad.osu.edu/admissions/quick-facts.html), our sample is less diverse than that of the U.S. population [77]. Second, males in our sample were under-represented; the small sample of males resulted in wide confidence intervals and reduced precision of the point estimates. With the reduced precision of the point estimates, the results for males should be interpreted with caution; with this said, our findings showing increased risk of disordered eating among dating violence-exposed males is consistent with the findings of prior studies [4,5]. Third, our first sample drawn from university registrar records had a response rate of 46.7%; we did not have information on non-responders to assess potential response bias. While our response rate of 46.7% may appear low, it is higher than other surveys of randomly-sampled adolescents (33%) [78]. In a meta-analysis, Cook and colleagues (2000) determined that the mean response rate for mostly paper surveys across 49 studies was 39.6% [79]. In a more recent meta-analysis comparing web- versus mail-survey modes, Shih and Fan (2008) reported that web survey modes generally have lower response rates (about 10% lower) than mail surveys [80]. Fourth, even with our detailed retrospective dating violence assessment approach and with our validation analyses of the dating violence questions [64,65], it is possible that subjects misestimated dating violence experiences [58]. This said, within the abuse assessment literature, variations in reporting are expected depending on how and when abuse is assessed [81-83]. In Jouriles’ study, differential abuse reporting was observed by differing recall periods, with subjects reporting higher rates of abuse in an ongoing reporting versus a retrospective reporting approach [59]. This suggests that as time passes, subjects may be less likely to report and/or remember abuse. If the same trends were to hold in our study which used a retrospective assessment approach, it is possible that some “exposed” teens were included in the “non-exposed” group, which would dilute our study findings. Fifth, because our survey was cross-sectional; it was not possible to assess temporality (directionality between violence and health). Moreover, the small sample size precluded a specific examination of health impacts by the timing of abuse (middle- versus late- adolescence), as we have done in our larger studies of adults [24,26,55]; future studies should examine these issues. Finally, there could be unmeasured confounding in our study; in our multivariable analyses, we controlled for other potentially traumatic exposures such as bullying and non-dating abuse suffered before age 18, but were unable in our survey to assess the full range of factors (e.g., parental substance abuse) that could introduce confounding.

Traumatic experiences during childhood adversely affect health in adulthood [58,72,73,84-89]. Exner-Cortens’ recent study showed that impaired health also results in adulthood from abuse experienced in adolescent relationships [16]. As an extension of the study’s limitations, we did not assess factors that could help explain pathways between dating violence and health. In the groundbreaking Adverse Childhood Experiences (ACE) studies, researchers suggest that the amplified health effects resulting from multiple adverse experiences in childhood, including violence, are due to the exposure of the brain to the stress response, which impairs brain structure and function [88,89]. We did not have information about factors, such as stress, that could fall in the pathway between dating violence and health. We are aware of studies currently under review that examine the impact of dating violence on stress responses; these studies will be helpful in the future for explaining pathways. Finally, our future work focuses on collecting qualitative information about adolescents’ dating violence experiences, including events leading up the abuse, abuse as it unfolds, and the aftermath. Information from forthcoming studies on stress responses and the qualitative nature of dating violence will further add to our understanding of dating violence.

## Conclusions

These limitations notwithstanding, our findings showed adverse health in adolescents exposed to physical/sexual and non-physical types of dating violence, particularly for victimized females. This highlights the need to implement programs, such as Safe Dates [61,90], to prevent dating violence and to intervene when it occurs.

## Abbreviations

CDC: Centers for disease control and prevention; CI: Confidence interval; OR: Odds ratio; PR: Prevalence ratio; TDV: Teen dating violence.

## Competing interest

The authors declare they have no competing financial or non-financial interests.

## Authors’ contributions

AEB conceptualized the study and survey, oversaw data collection and analysis, and wrote the manuscript with co-authors’ input. MA helped design the survey, conducted the data analysis, and critically reviewed the manuscript. JMN and CB helped conceptualize the study and survey, added to the statistical analysis, and critically reviewed the manuscript. FPR helped design the survey and critically reviewed the manuscript. All authors read and approved the final manuscript.

## Pre-publication history

The pre-publication history for this paper can be accessed here:

http://www.biomedcentral.com/1471-2458/13/821/prepub
